# Supplementary material for: Predicting visual function by interpreting a neuronal wiring diagram
Source: Nature. 2024 Oct 2;634(8032):113–23. doi: 10.1038/s41586-024-07953-5 (PMC11446822; doi:10.1038/s41586-024-07953-5)
Supplement: Supplementary file 6 — CRF and ERF predictions for individual TmY4 and TmY9 cells. Analogous to Supplementary Data 3, but for TmY target types. Shown are the top four monosynaptic pathways, the strongest pathway passing through each of the top ten intermediary types (ranking from Extended Data Fig. 7), and the trisynaptic pathway Tm1–TmY–Dm3–TmY (see the section entitled Prediction of spatial normalization). [file 41586_2024_7953_MOESM6_ESM.zip › DataS4/TmY9qΓèÑ/720575940612523506.pdf]

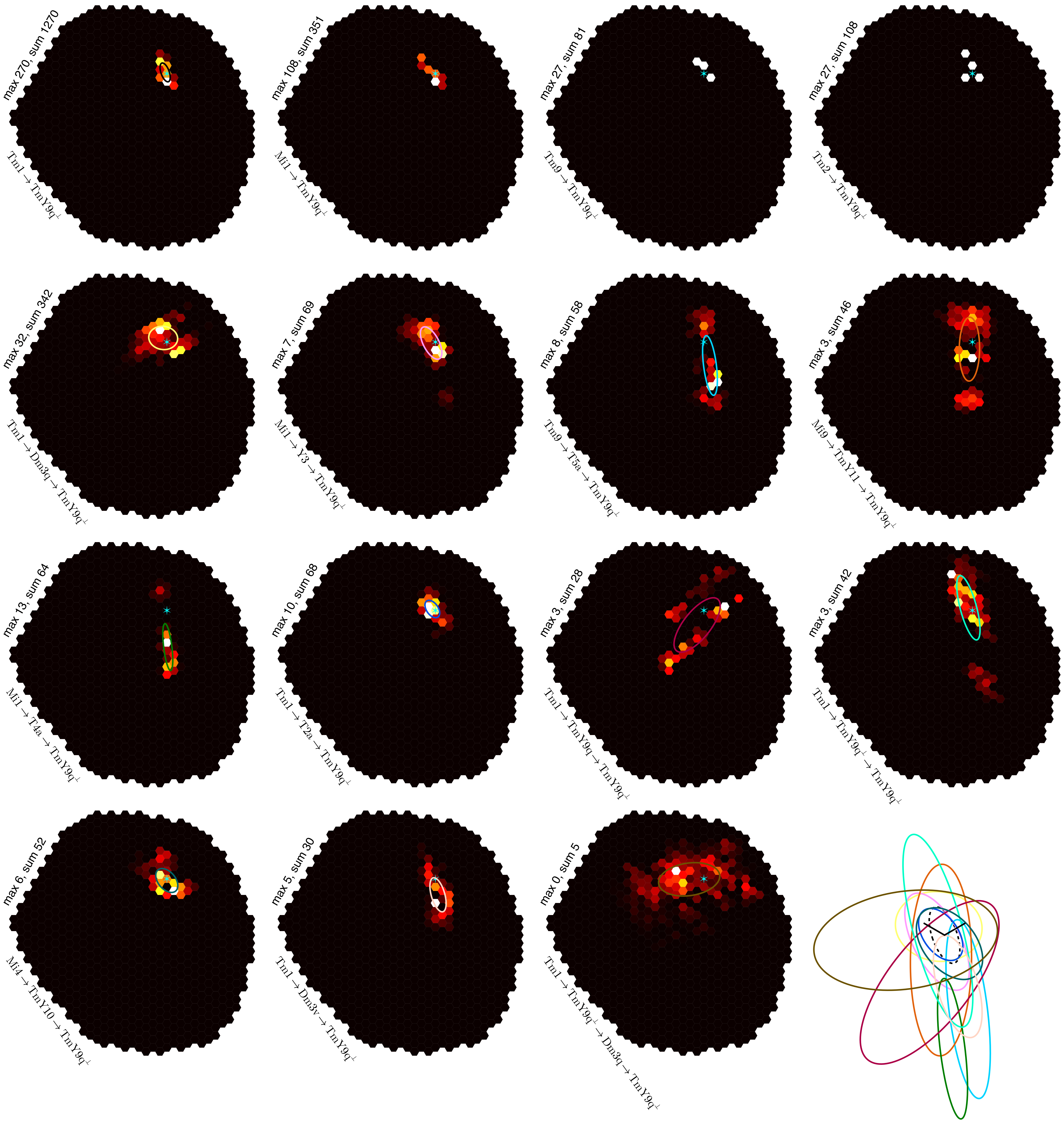
max 270, sum 1270
Tm1 → TmY9q⊥
max 108, sum 351
Mi1 → TmY9q⊥
max 27, sum 81
Tm9 → TmY9q⊥
max 27, sum 108
Tm2 → TmY9q⊥
max 32, sum 342
Tm1 → Dm3q → TmY9q⊥
max 7, sum 69
Mi1 → Y3 → TmY9q⊥
max 8, sum 58
Tm9 → T5a → TmY9q⊥
max 3, sum 46
Mi9 → TmY11 → TmY9q⊥
max 13, sum 64
Mi1 → T4a → TmY9q⊥
max 10, sum 68
Tm1 → T2a → TmY9q⊥
max 3, sum 28
Tm1 → TmY9q → TmY9q⊥
max 3, sum 42
Tm1 → TmY9q⊥ → TmY9q⊥
max 6, sum 52
Mi4 → TmY10 → TmY9q⊥
max 5, sum 30
Tm1 → Dm3v → TmY9q⊥
max 0, sum 5
Tm1 → TmY9q⊥ → Dm3q → TmY9q⊥
